# Supplementary material for: Short 2′-O-methyl/LNA oligomers as highly-selective inhibitors of miRNA production in vitro and in vivo
Source: Nucleic Acids Res. 2024 Apr 27;52(10):5804–24. doi: 10.1093/nar/gkae284 (PMC11162791; doi:10.1093/nar/gkae284)
Supplement: gkae284_Supplemental_Files [file gkae284_supplemental_files.zip › Corradi_et_al_SI_Rev2.pdf]

Supplementary information for

Short 2′-O-methyl/LNA oligomers as highly-selective inhibitors of miRNA production *in vitro* and *in vivo*

Natalia Koralewska†, Eloina Corradi†, Marek C. Milewski, Linda Masante, Agnieszka Szczepanska, Ryszard Kierzek, Marek Figlerowicz, Marie-Laure Baudet*, Anna Kurzynska-Kokorniak*

† Joint Authors
* To whom correspondence should be addressed. Anna Kurzynska-Kokorniak; Email: akurzyns@man.poznan.pl. Correspondence may also be addressed to Marie-Laure Baudet; Email: marielaure.baudet@unitn.it.

This PDF file includes the following:

Supplementary Figures S1 to S11

Legend for Supplementary Table S1

Supplementary Tables S2 to S4

Other supplementary materials for this manuscript include:

Supplementary Table S1

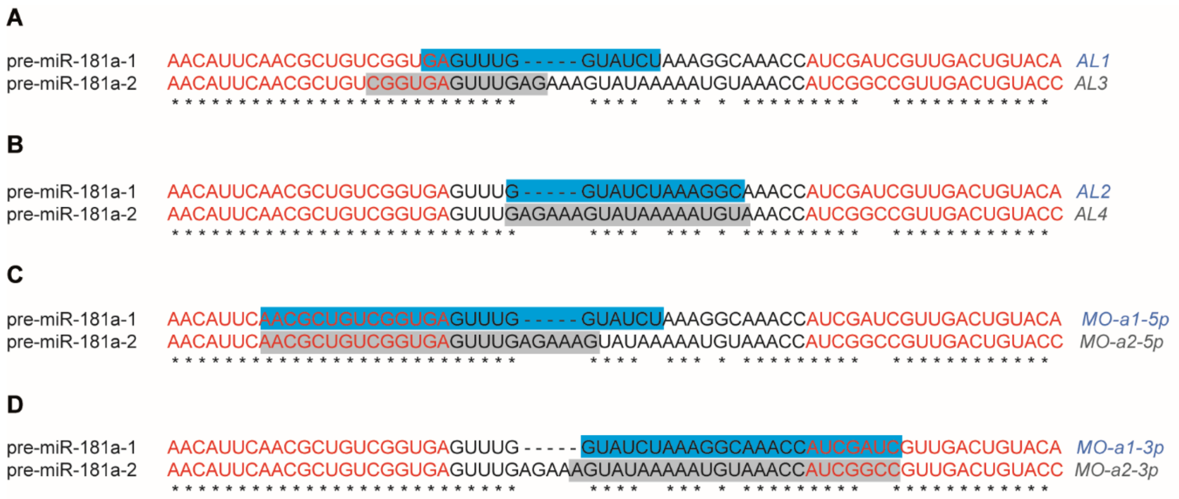

Supplementary Figure S1. The comparison of the identity of target sequences for all inhibitors used in the study

(A-D) The alignment of pre-miR-181a-1 and pre-miR-181a-2 generated in Clustal Omega. Target sites for (A) AL1 and AL3, (B) AL2 and AL4, (C) MOs-5p, (D) MOs-3p are marked with the highlights. Red font indicates the sequences of miRNAs.

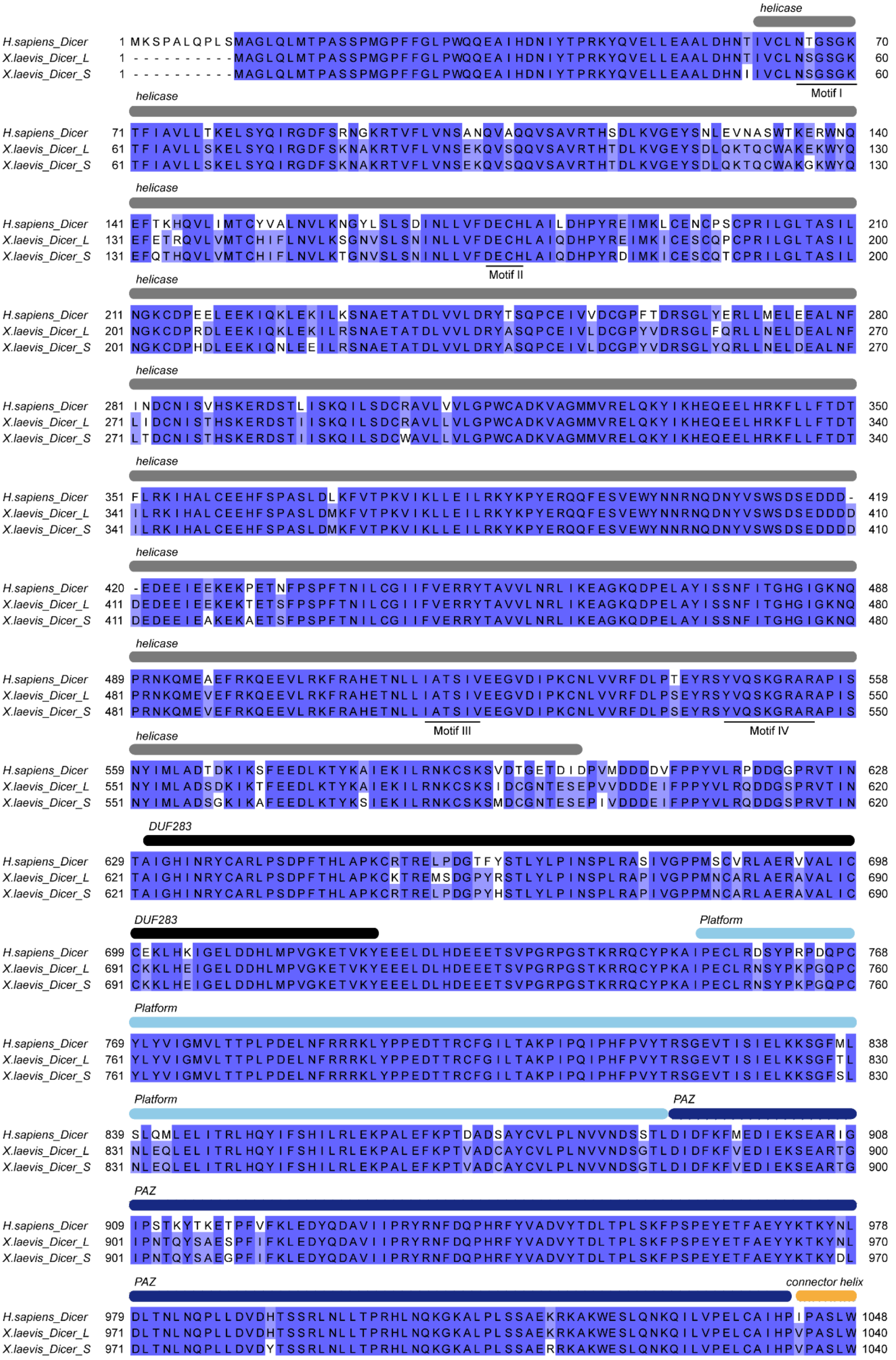
helicase
H.sapiens_Dicer 1 MKSPALQPLSMAGLQLMTPASSPMGPFFGLPWQQEAIHDNIYTPRKYQVELLEAALDHNTIVCLNTGSGK 70
X.laevis_Dicer_L 1 ----------MAGLQLMTPASSPMGPFFGLPWQQEAIHDNIYTPRKYQVELLEAALDHNTIVCLNSGSGK 60
X.laevis_Dicer_S 1 ----------MAGLQLMTPASSPMGPFFGLPWQQEAIHDNIYTPRKYQVELLEAALDHNIIVCLNSGSGK 60
Motif I
helicase
H.sapiens_Dicer 71 TFIAVLLTKELSYQIRGDFSRNGKRTVFLVNSANQVAQQVSAVRTHSDLKVGEYSNLEVNASWTKERWNQ 140
X.laevis_Dicer_L 61 TFIAVLLSKELSYQIRGDFSKNAKRTVFLVNSEKQVSQQVSAVRTHTDLKVGEYSDLQKTQCWAKEKWYQ 130
X.laevis_Dicer_S 61 TFIAVLLSKELSYQIRGDFSKNAKRTVFLVNSEKQVSQQVSAVRTHTDLKVGEYSDLQKTQCWAKGKWYQ 130
helicase
H.sapiens_Dicer 141 EFTKHQVLIMTCYVALNVLKNGYLSLSDINLLVFDECHLAILDHPYREIMKLCENCPSCPRILGLTASIL 210
X.laevis_Dicer_L 131 EFETRQVLVMTCHIFLNVLKSGNVSLSNINLLVFDECHLAIQDHPYREIMKICESCQPCPRILGLTASIL 200
X.laevis_Dicer_S 131 EFQTHQVLVMTCHIFLNVLKTGNVSLSNINLLVFDECHLAIQDHPYRDIMKICESCQTCPRILGLTASIL 200
Motif II
helicase
H.sapiens_Dicer 211 NGKCDPEELEEKIQKLEKILKSNAETATDLVVLDRYTSQPCEIVVDCGPFTDRSGLYERLLMELEEALNF 280
X.laevis_Dicer_L 201 NGKCDPRDLEEKIQKLEKILRSNAETATDLVVLDRYASQPCEIVLDCGPYVDRSGLFQRLLNELDEALNF 270
X.laevis_Dicer_S 201 NGKCDPHDLEEKIQNLEEILRSNAETATDLVVLDRYASQPCEIVLDCGPYVDRSGLYQRLLNELDEALNF 270
helicase
H.sapiens_Dicer 281 INDCNISVHSKERDSTLISKQILSDCRAVLVVLGPWCADKVAGMMVRELQKYIKHEQEELHRKFLLFTDT 350
X.laevis_Dicer_L 271 LIDCNISTHSKERDSTIISKQILSDCRAVLLVLGPWCADKVAGMMVRELQKYIKHEQEELHRKFLLFTDT 340
X.laevis_Dicer_S 271 LTDCNISTHSKERDSTIISKQILSDCWAVLLVLGPWCADKVAGMMVRELQKYIKHEQEELHRKFLLFTDT 340
helicase
H.sapiens_Dicer 351 FLRKIHALCEEHFSPASLDLKFVTPKVIKLLEILRKYKPYERQQFESVEWYNNRNQDNYVSWSDSEDDD- 419
X.laevis_Dicer_L 341 ILRKIHALCEEHFSPASLDMKFVTPKVIKLLEILRKYKPYERQQFESVEWYNNRNQDNYVSWSDSEDDDD 410
X.laevis_Dicer_S 341 ILRKIHALCEEHFSPASLDMKFVTPKVIKLLEILRKYKPYERQQFESVEWYNNRNQDNYVSWSDSEDDDD 410
helicase
H.sapiens_Dicer 420 -EDEEIEEKEKPETNFPSPFTNILCGIIFVERRYTAVVLNRLIKEAGKQDPELAYISSNFITGHGIGKNQ 488
X.laevis_Dicer_L 411 DEDEEIEEKEKTETSFPSPFTNILCGIIFVERRYTAVVLNRLIKEAGKQDPELAYISSNFITGHGIGKNQ 480
X.laevis_Dicer_S 411 DEDEEIEAKEKAETSFPSPFTNILCGIIFVERRYTAVVLNRLIKEAGKQDPELAYISSNFITGHGIGKNQ 480
helicase
H.sapiens_Dicer 489 PRNKQMEAEFRKQEEVLRKFRAHETNLLIATSIVEEGVDIPKCNLVVRFDLPTEYRSYVQSKGRARAPIS 558
X.laevis_Dicer_L 481 PRNKQMEVEFRKQEEVLRKFRAHETNLLIATSIVEEGVDIPKCNLVVRFDLPSEYRSYVQSKGRARAPIS 550
X.laevis_Dicer_S 481 PRNKQMEVEFRKQEEVLRKFRAHETNLLIATSIVEEGVDIPKCNLVVRFDLPSEYRSYVQSKGRARAPIS 550
Motif III
Motif IV
helicase
H.sapiens_Dicer 559 NYIMLADTDKIKSFEEDLKTYKAIEKILRNKCSKSVDTGETDIDPVMDDDDVFPPYVLRPDDGGPRVTIN 628
X.laevis_Dicer_L 551 NYIMLADSDKIKTFEEDLKTYKAIEKILRNKCSKSIDCGNTESEPVVDDDEIFPPYVLRQDDGSPRVTIN 620
X.laevis_Dicer_S 551 NYIMLADSGKIKAFEEDLKTYKSIEKILRNKCSKSMDCGNTESEPIVDDDEIFPPYVLRQDDGSPRVTIN 620
DUF283
H.sapiens_Dicer 629 TAIGHINRYCARLPSDPFTHLAPKCRTRELPDGTFYSTLYLPINSPLRASIVGPPMSCVRLAERVVALIC 698
X.laevis_Dicer_L 621 TAIGHINRYCARLPSDPFTHLAPKCKTREMSDGPYRSTLYLPINSPLRAPIVGPPMNCARLAERAVALIC 690
X.laevis_Dicer_S 621 TAIGHINRYCARLPSDPFTHLAPKCRTRELPDGPYHSTLYLPINSPLRAPIVGPPMNCARLAERAVALIC 690
DUF283
Platform
H.sapiens_Dicer 699 CEKLHKIGELDDHLMPVGKETVKYEEELDLHDEEETSVPGRPGSTKRRQCYPKAIPECLRDSYPRPDQPC 768
X.laevis_Dicer_L 691 CKKLHEIGELDDHLMPVGKETVKYEEELDLHDEEETSVPGRPGSTKRRQCYPKAIPECLRNSYPKPGQPC 760
X.laevis_Dicer_S 691 CKKLHEIGELDDHLMPVGKETVKYEEELDLHDEEETSVPGRPGSTKRRQCYPKAIPECLRNSYPKPGQPC 760
Platform
H.sapiens_Dicer 769 YLYVIGMVLTTPLPDELNFRRRKLYPPEDTTRCFGILTAKPIPQIPHFPVYTRSGEVTISIELKKSGFML 838
X.laevis_Dicer_L 761 YLYVIGMVLTTPLPDELNFRRRKLYPPEDTTRCFGILTAKPIPQIPHFPVYTRSGEVTISIELKKSGFTL 830
X.laevis_Dicer_S 761 YLYVIGMVLTTPLPDELNFRRRKLYPPEDTTRCFGILTAKPIPQIPHFPVYTRSGEVTISIELKKSGFSL 830
Platform
PAZ
H.sapiens_Dicer 839 SLQMLELITRLHQYIFSHILRLEKPALEFKPTDADSAYCVLPLNVVNDSSTLDIDFKFMEDIEKSEARIG 908
X.laevis_Dicer_L 831 NLEQLELITRLHQYIFSHILRLEKPALEFKPTVADCAYCVLPLNVVNDSGTLDIDFKFVEDIEKSEARTG 900
X.laevis_Dicer_S 831 NLEQLELITRLHQYIFSHILRLEKPALEFKPTVADCAYCVLPLNVVNDSGTLDIDFKFVEDIEKSEARTG 900
PAZ
H.sapiens_Dicer 909 IPSTKYTKETPFVFKLEDYQDAVIIPRYRNFDQPHRFYVADVYTDLTPLSKFPSPEYETFAEYYKTKYNL 978
X.laevis_Dicer_L 901 IPNTQYSAESPFIFKLEDYQDAVIIPRYRNFDQPHRFYVADVYTDLTPLSKFPSPEYETFAEYYKTKYNL 970
X.laevis_Dicer_S 901 IPNTQYSAEGPFIFKLEDYQDAVIIPRYRNFDQPHRFYVADVYTDLTPLSKFPSPEYETFAEYYKTKYDL 970
PAZ
connector helix
H.sapiens_Dicer 979 DLTNLNQPLLDVDHTSSRLNLLTPRHLNQKGKALPLSSAEKRKAKWESLQNKQILVPELCAIHPIPASLW 1048
X.laevis_Dicer_L 971 DLTNLNQPLLDVDHTSSRLNLLTPRHLNQKGKALPLSSAEKRKAKWESLQNKQILVPELCAIHPVPASLW 1040
X.laevis_Dicer_S 971 DLTNLNQPLLDVDYTSSRLNLLTPRHLNQKGKALPLSSAERRKAKWESLQNKQILVPELCAIHPVPASLW 1040

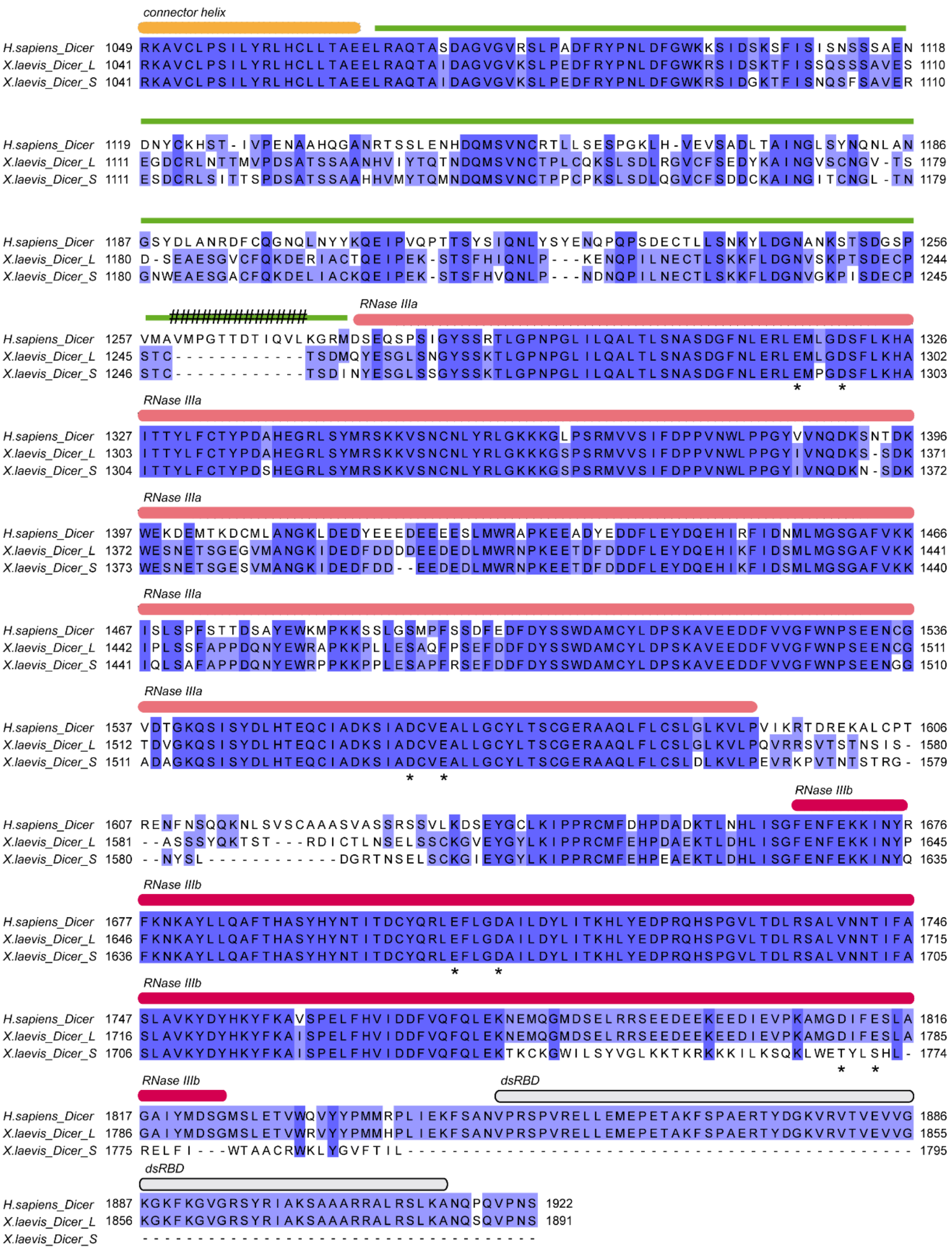

Supplementary Figure S2. The comparison of the sequences of human and *X. laevis* Dicer proteins

Multiple sequence alignment performed using ClustalW [1]. Sequences retrieved from UniProtKB: Q9UPY3, A0A1L8F9L1, D0UED5. Graded blue highlights of the sequence indicate the level of amino acid conservation. Protein domains are marked above the sequences. Colour code as in Figure 2. Asterisks (*) indicate amino acids of the catalytic core; hashtags (#) point a 12-aa difference within the molecular ruler helix among human and *X. laevis* proteins. Abbreviations: DUF283, domain of unknown function 283; PAZ, Piwi/Argonaute/Zwille domain; dsRBD, dsRNA binding domain.

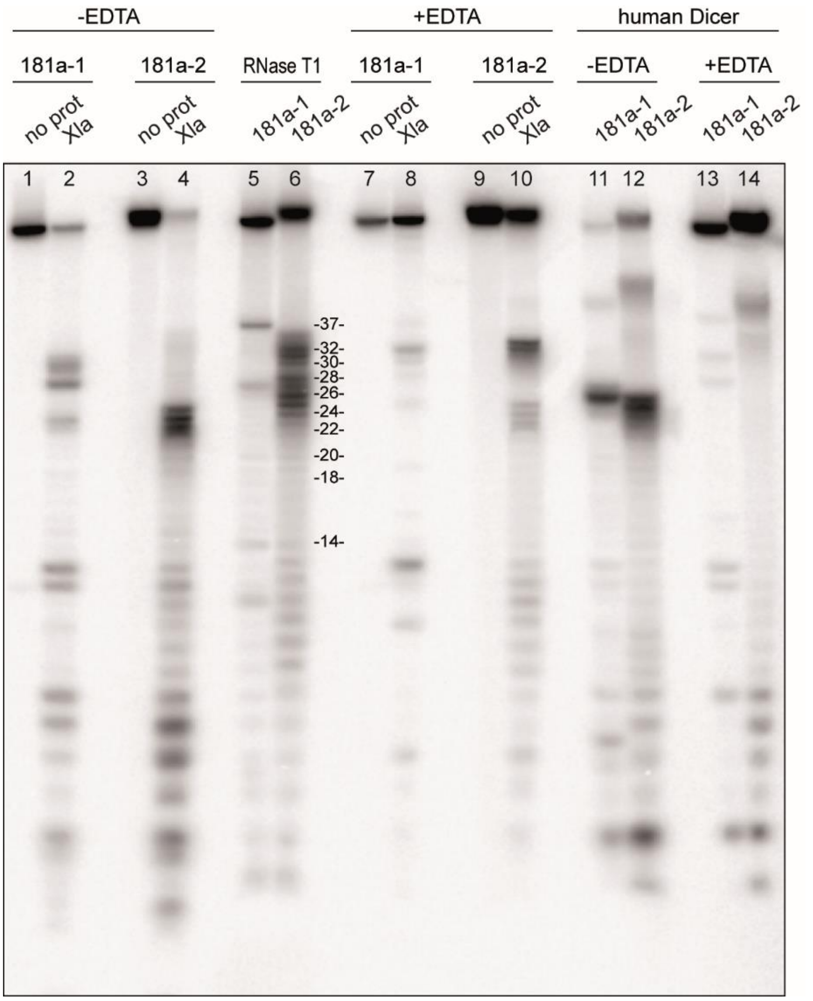

Supplementary Figure S3. Characterization of the pre-miRNA cleavage pattern generated in cytosolic extracts

Uncropped image of a representative gels presented in Figure 2, showing the full range of products generated upon the incubation of 5′-^{32}P-labeled pre-miRNA with *Xenopus* cytosolic extracts (lines 1-4, 7-10) or human Dicer (lines 11-14), with or without EDTA. Partial hydrolysis of RNA by RNase T1 was used to generate a ladder (lines 5, 6). Reproducible results were obtained using different batches of the extracts.

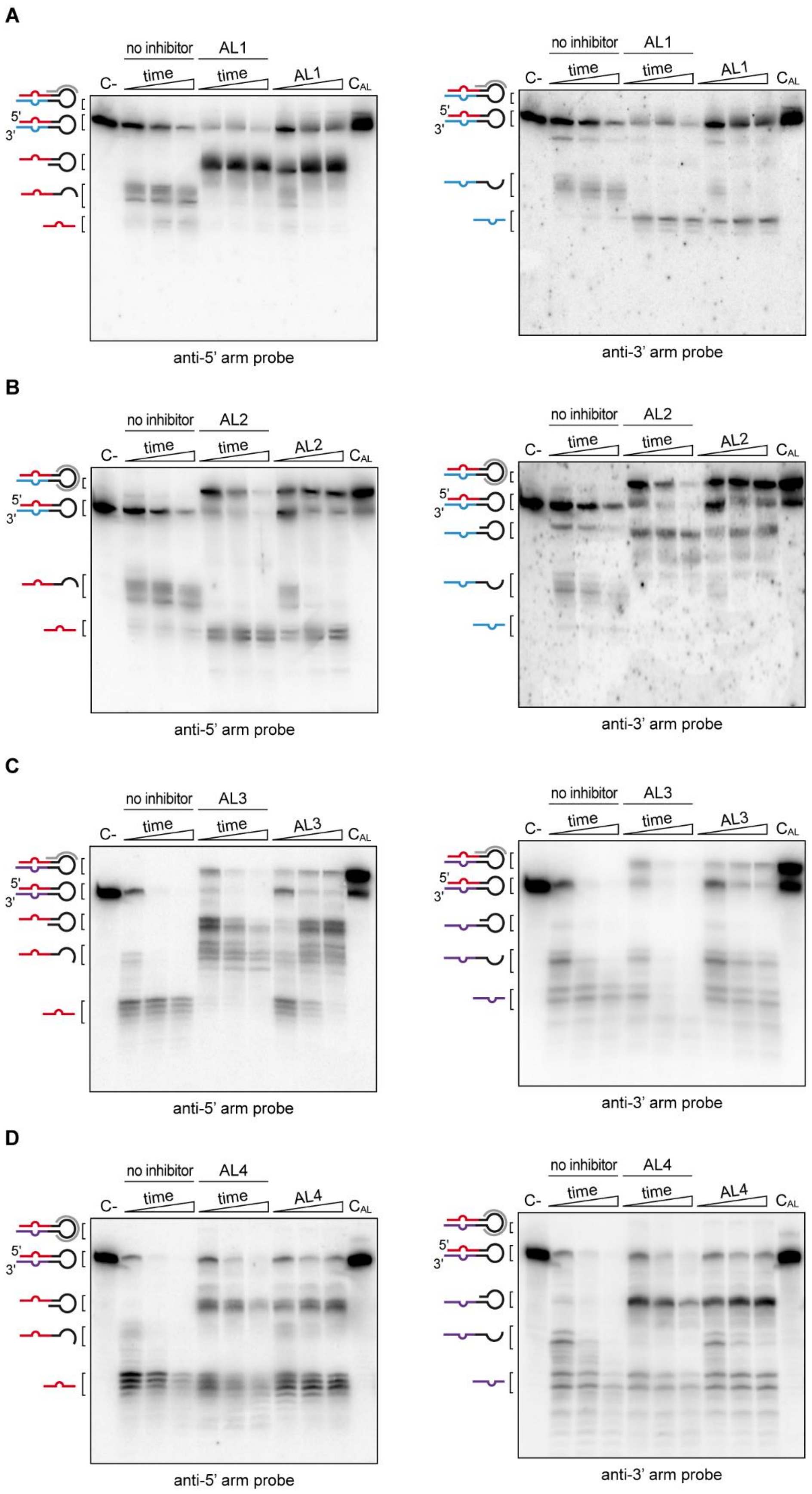

Supplementary Figure S4. Validation of pre-miR-181a-1 and pre-miR-181a-2 cleavage pattern in the presence of 2'-OMe/LNA ASOs targeting 5' arm of the precursors

(A-D) Northern blot analysis of the cleavage pattern of pre-miR-181a-1 in the presence of AL1 (A) or AL2 (B), and pre-miR-181a-2 in the presence of AL3 (C) or AL4 (D). RNA was incubated

with 100 molar excess of the indicated AL and *Xenopus* cytosolic extract for 0.5, 1.5, 3 h (time change indicated by a triangle) or with 1, 10 or 100 molar excess of the given AL for 0.5 h (AL concentration change indicated by a triangle). RNA was visualized using DNA probes targeting either 5′ or 3′ arm of the pre-miRNA as indicated. Schematic representation of the pre-miRNA, AL and cleavage products is given on the left to the blots. Abbreviations: C-, pre-miRNA incubated in a buffer with no AL nor protein; C_{AL}, pre-miRNA incubated in a buffer with AL but without protein.

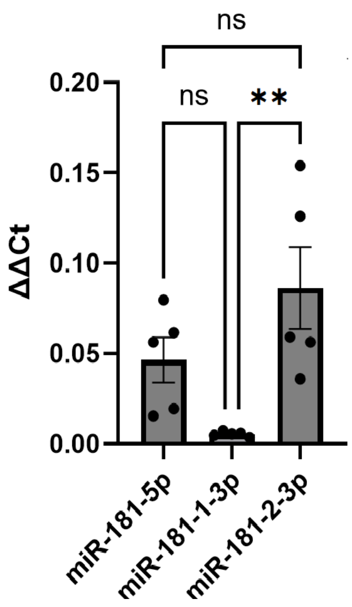

Supplementary Figure S5. Relative abundance of miRNA in the eyes of WT stage 40 *X. laevis* embryos

miRNAs expression levels quantified using the $2^{-\Delta Ct}$ method and U6 as normalizer. Data information: Values are calculated using the data from the experiments presented in Figure 4 and Supplementary Figure S8. Values are mean ± SEM. Statistics: n=5 independent experiments, each data point represents a single RT–qPCR, 1-way Anova, Tukey's multiple comparisons test. Abbreviations: ns, not significant.

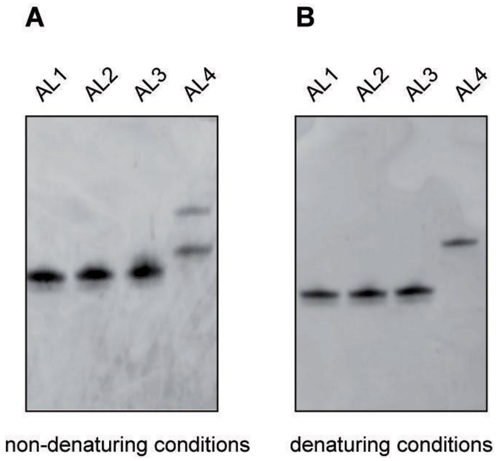

Supplementary Figure S6. Assessment of RNA secondary structure forms adopted by designed 2′-OMe/LNA oligomers

(A) RNA folding analyzed by non-denaturing PAGE of the unlabeled 2′-OMe/LNA antisense oligomers (AL1-4), followed by SYBR Gold staining. **(B)** RNA integrity verified by denaturing PAGE of the unlabeled 2′-OMe/LNA ASOs (AL1-4), followed by SYBR Gold staining.

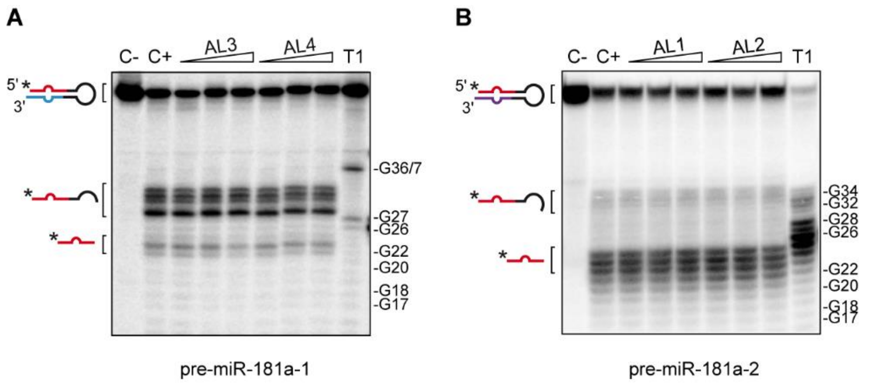

Supplementary Figure S7. *In vitro* validation of the target-specificity of the designed 2'-OMe/LNA ASOs

(A, B) 5'-^{32}P-labeled pre-miR-181a-1 (A) and pre-miR-181a-2 (B) were incubated with *Xenopus* cytosolic extract in the presence of AL3 or AL4, and AL1 or AL2, as indicated. Triangles represent increasing amounts of the indicated AL (pre-miRNA:oligomer molar ratios of 1:1, 1:10, and 1:100). Abbreviations: C-, sample with no cytosolic extract, nor inhibitor added; C+, sample with *Xenopus* cytosolic extract, without any inhibitor; T1, RNase T1 ladder.

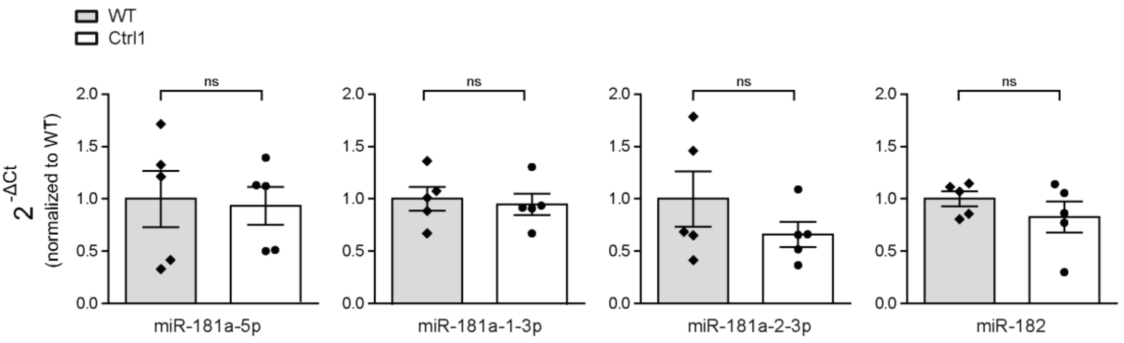

Supplementary Figure S8. *In vivo* evaluation of 2′-OMe/LNA control oligomer

miRNAs expression levels quantified using the $2^{-\Delta Ct}$ method and U6 as normalizer, after Ctrl oligomer microinjection. Data are normalized to WT control embryos. Data information: Values are mean ± SEM. Statistics: n=5 independent experiments, each data point represents a single RT–qPCR, unpaired t-test. Abbreviations: WT, wild type; ns, not significant.

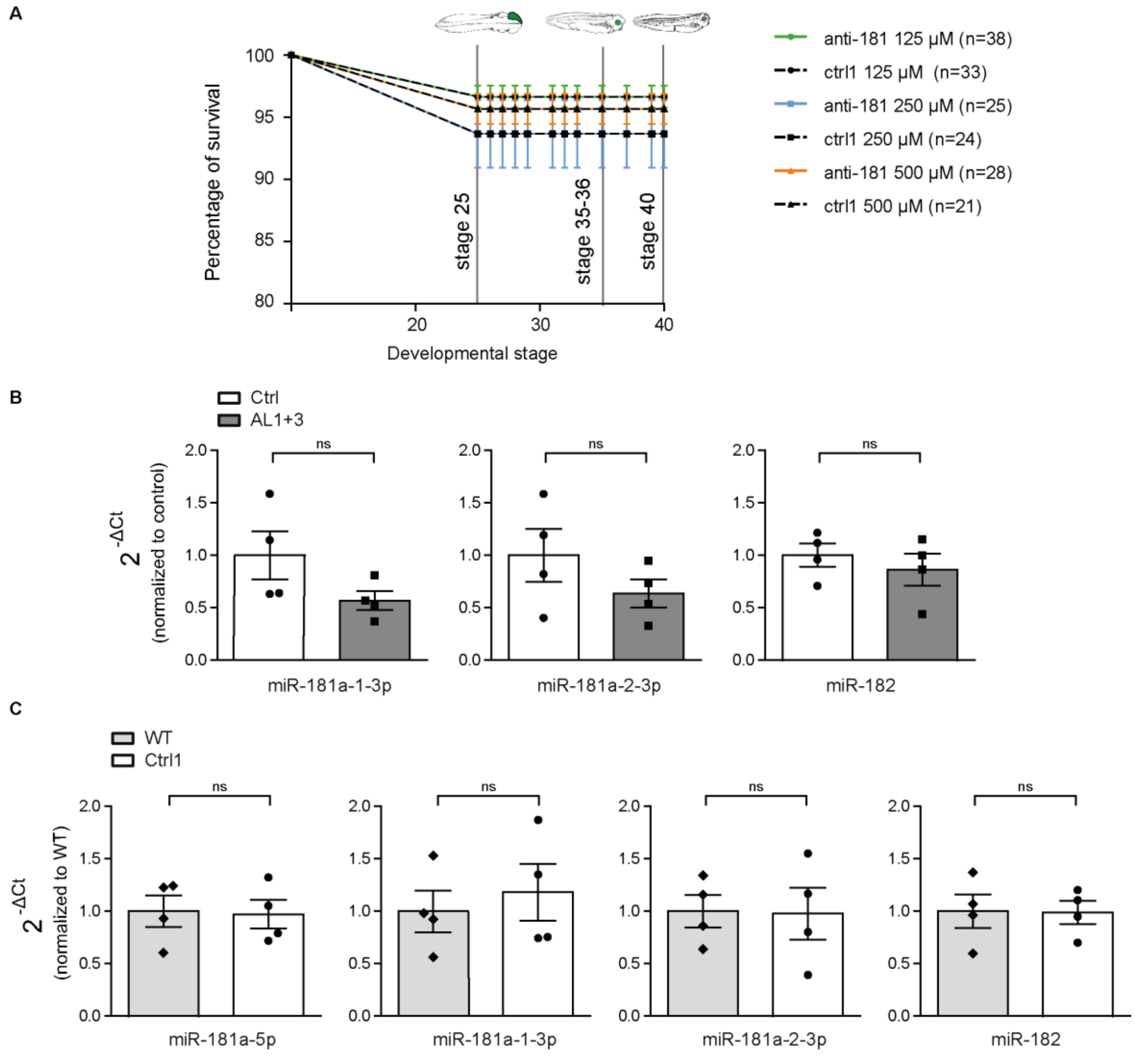

Supplementary Figure S9. *In vivo* evaluation of 2´-OMe/LNA ASOs compatibility

(A) Survival embryos rate after inhibitors eye delivery, in controls and AL1+3. **(B, C)** miRNAs expression levels quantified using the $2^{-\Delta Ct}$ method and U6 as normalizer. Data are normalized to control (B) or to WT (C). Data information: Values are mean ± SEM. Statistics: n=3 independent experiments for each of the concentrations tested (A), total number of embryos is reported in parenthesis (A); n=5 independent experiments, each data point represents a single RT–qPCR, unpaired t-test (B, C). Abbreviations: ns, not significant; WT, wild type.

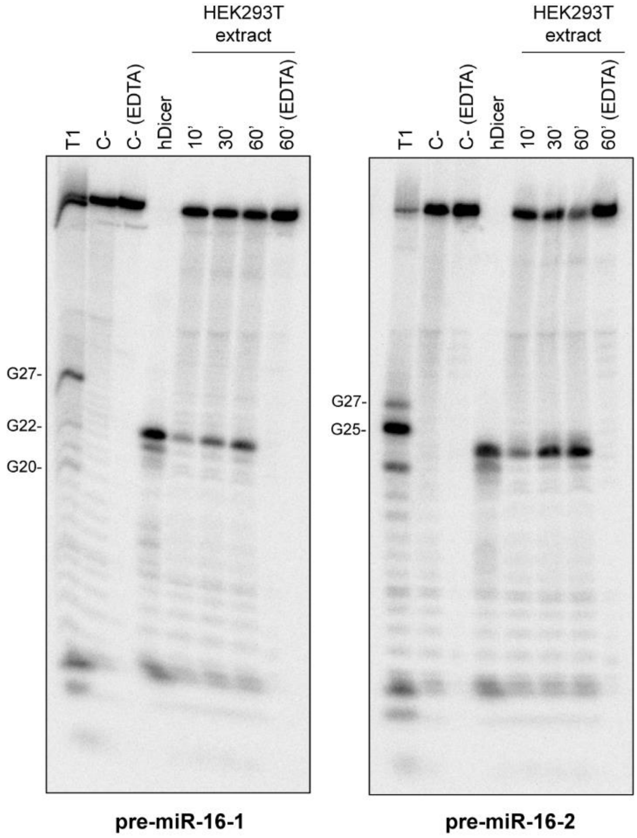

Supplementary Figure S10. Validation of pre-miRNA processing by human Dicer present in HEK293T cytosolic extracts

Pre-miR-16-1 and pre-miR-16-2 were incubated with human Dicer for 1 h or with HEK293T cytosolic extracts for 10 min, 30 min or 60 min. The ladder was created based on pre-miR-16-1 and pre-miR-16-2 cleavage patterns generated by RNase T1. C-, a control reaction with no extract or protein added, (EDTA), reaction mixtures supplemented with 25 mM EDTA.

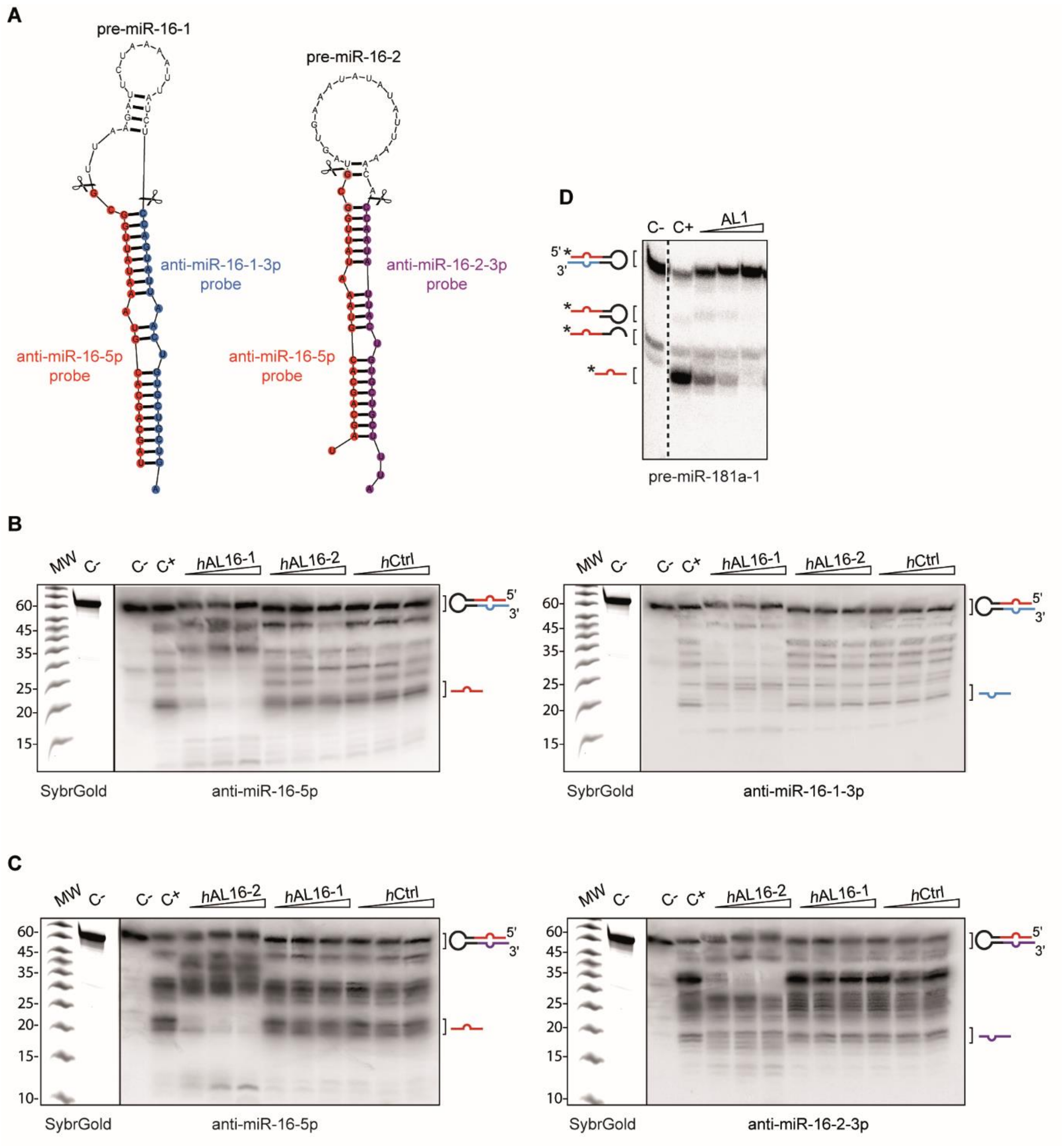

Supplementary Figure S11. Validation of 2′-OMe/LNA ASOs arm-selectivity in HEK293T cytosolic extracts model

(A) Schematic representation of human pre-miR-16-1 and pre-miR-16-2 structures, regions targeted by probes used in Northern blot assays are indicated. **(B, C)** Northern blot analysis of the cleavage pattern of pre-miR-16-1 (B) and pre-miR-16-2 (C) in the presence of *h*AL1, *h*AL2 and control oligomer *h*Ctrl. RNA was incubated with 1, 10 or 100 molar excess of the given oligomer for 1 h (oligomer concentration change indicated by a triangle). RNA was visualized using DNA probes targeting either 5′ or 3′ arm of the pre-miRNA as indicated. **(D)** Processing of *X. laevis* pre-miR-181a-1 by human Dicer in the presence of AL1 was tested using 5′-^{32}P-labeled pre-miRNA and HEK293T cytosolic extracts. A triangle indicates increasing amount of AL1. Schematic representation of pre-miRNA and cleavage products is indicated next to the scan image. Abbreviations: C-, pre-miRNA incubated in a buffer with no AL nor protein; C+, pre-miRNA incubated in extracts without AL/Ctrl, MW, small RNA ladder (FUTUREsynthesis).

Supplementary Table S1. Characterization of the interaction between AL-ASOs designed in the study and potential targets found within *X. laevis* miRNA and pre-miRNA (a separate Excel file)

Supplementary Table S2. Sequences (5′-3′) of the best 162 negative control oligomers that can be used in *X. laevis* model. The oligomer used in the study is marked in red.

1 CGUAUACUUCGCG	53 CUACCGCGUAAGG	105 GUUAGGUCGCGUA	157 UCGCGUACCGAGU
2 UCGCGCCACGAUA	54 CCUUACGCGGUAG	106 CGUCGAUACUAGG	158 ACUCGGUACGCGA
3 UAUCGUGGCGCGA	55 ACGCCUAGCGUAA	107 CCUAGUAUCGACG	159 UUACGCGCCGUAA
4 CUACGCGGCGUAA	56 UACGCGAAUCGUU	108 UACGCGAUCCGAU	160 UUACGGCGCGUAA
5 UACGCGGCGUAAC	57 AACGAUUCGCGUA	109 AUCGGAUCGCGUA	161 UCGCGGAGUACUA
6 GUUACGCCGCGUA	58 UAAUCGCGUACCG	110 CGCGACCUAUCGU	162 UAGUACUCCGCGA
7 CUCGCGAAUAACG	59 CGGUACGCGAUUA	111 ACGAUAGGUCGCG	
8 UCGCGUAUUCGCG	60 UUAGCGCGGUACG	112 GUUCGUAACGCGC	
9 CGCGAAUACGCGA	61 CGUACCGCGCUAA	113 GCGCGUUACGAAC	
10 UACGCGCAUCGUA	62 UUCGACGCGAGUA	114 CGUUCGCGUAGGU	
11 UAUUACGCGAGCG	63 UACUCGCGUCGAA	115 ACCUACGCGAACG	
12 CGCUCGCGUAAUA	64 UAAGUCGACGCGU	116 CUACGUACGCUCG	
13 UUACGUUACGCGA	65 UACGACGGUAAGC	117 UCGGCGUAGUACC	
14 UCGCGUAACGUAA	66 ACGCGUCGACUUA	118 GGUACUACGCCGA	
15 CUAUCGCGCUAGU	67 GCUUACCGUCGUA	119 GUUACGAUCGCGC	
16 CGCGAAUUAAUCG	68 UCGUACGCGAUUG	120 UUCGUACCGCUAG	
17 CGUUAGUACGCCG	69 CGUCGUUAGGACG	121 CUAGCGGUACGAA	
18 CGGCGUACUAACG	70 CGUCCUAACGACG	122 UCGUUAGGUCGCG	
19 ACUAGCGCGAUAG	71 UAUCGACGCGUAG	123 CGGUCGUAUACCG	
20 UAAGCGCGUAACG	72 CUACGCGUCGAUA	124 CGGUAUACGACCG	
21 CGUUACGCGCUUA	73 CAAUCGCGUACGA	125 CGCGACCUAACGA	
22 CGUAACGAACCGU	74 CGUCGUCGCGAAU	126 GCACGCUAGUACG	
23 CCGCGUAGCGUAU	75 AUUCGCGACGACG	127 CGUACUAGCGUGC	
24 AUACGCUACGCGG	76 GCUAGUCUACGCG	128 CGAUACCGUACGC	
25 ACGGUUCGUUACG	77 CGCGUAGACUAGC	129 GGACGUACGCGAU	
26 UUACGCGACGCGU	78 CGCGAUACGAUCU	130 CGACUAACGGUCG	
27 CUAUCUACGCUCG	79 AGAUCGUAUCGCG	131 CGACCGUUAGUCG	
28 CGAGCGUAGAUAG	80 GUCGCGUACCGUU	132 CGUCGAUACGUCG	
29 ACGCGUCGCGUAA	81 CGCCGUACGUAAU	133 CGACGUAUCGACG	
30 UUACGCGAACCUA	82 AUUACGUACGGCG	134 CGUUCGCGUAUCG	
31 UAGGUUCGCGUAA	83 AACGGUACGCGAC	135 CGAUACGCGAACG	
32 UUACGCCGCGUAG	84 UCGCGCAAUCUUA	136 CUAAUUCGCGACG	
33 CGUUCGCGUAAGU	85 GUUCGCGCCUAAG	137 CGUCGCGAAUUAG	
34 CUACCGAUUAGCG	86 CUUAGGCGCGAAC	138 GCGUACUACGACG	
35 ACUUACGCGAACG	87 UUAGUCGAGCGCG	139 CGUCGUAGUACGC	
36 CGCUAAUCGGUAG	88 UACGGCGCGUAAC	140 UCACGAUUGCGCG	
37 UAACUAGCUCGCG	89 GUUACGCGCCGUA	141 CGCGCAAUCGUGA	
38 UAACUCGCGGACG	90 CGCGCUCGACUAA	142 CGCGGUCGAUAGU	
39 GUACGCGAGUACG	91 UAAGUUCGCGACG	143 ACUAUCGACCGCG	
40 CGUACUCGCGUAC	92 CGUCGCGAACUUA	144 GUCGAUCGACGAU	
41 CGUCCGCGAGUUA	93 UACGCGAAGUCCG	145 GUCGACAUCGCGU	
42 CGCGUACUACCGU	94 CGGACUUCGCGUA	146 UCGACUAACGUCG	
43 ACGGUAGUACGCG	95 UACUAGCGUACCG	147 CGACGUUAGUCGA	
44 CCGCGACUUAGUU	96 CGGUACGCUAGUA	148 UAUAUCGACGCGU	
45 AACUAAGUCGCGG	97 UACGCGACUAGCC	149 ACGCGUCGAUAUA	
46 UCGACCGCGUAAG	98 UGCGCGUCGAAUG	150 GUUACGCGAACCG	
47 CUUACGCGGUCGA	99 UUAACGGUACGCG	151 CGGUUCGCGUAAC	
48 UACGCGUCGAUAG	100 CAUUCGACGCGCA	152 ACUAGGUACGCGU	
49 CUAUCGACGCGUA	101 CGCGUACCGUUAA	153 ACGCGUACCUAGU	
50 CGGUUCGACGUAU	102 GUACGCGUUAGUU	154 UACUCGCGACCAC	
51 AUACGUCGAACCG	103 AACUAACGCGUAC	155 UAAGAUCGCGUAG	
52 UUACGCUAGGCGU	104 UACGCGACCUAAC	156 CUACGCGAUCUUA	

Supplementary Table S3. Parameters of the thermodynamic stability of pre-miR-181a-1 complexes with AL1 and AL2, and pre-miR-181a-2 complexes with AL3 and AL4[a]

		Average of curve fits				
		$-\Delta H^\circ$ (kcal/mol)	$-\Delta S^\circ$ (eu)	$-\Delta G^\circ_{37}$ (kcal/mol)	$T_M{}^b$ (°C)	
pre-miR-181a-1	AL1	74.3±11.7	214.4±37.3	7.80±0.19	42.4	Duplex
pre-miR-181a-1	AL1	124.6±10.6	364.7±33.2	11.49±0.19	68.5	Hairpin
pre-miR-181a-1	AL2	83.9±9.4	240.6±30.4	9.30±0.45	47.6	Duplex
pre-miR-181a-1	AL2	92.6±1.6	262.1±4.9	11.35±0.55	80.3	Hairpin
pre-miR-181a-1		106.6±5.4	323.8±16.5	6.19±0.38	56.1	Hairpin
pre-miR-181a-2	AL3	56.4±7.4	154.5±22.4	8.50±0.48	48.2	Duplex
pre-miR-181a-2	AL3	112.8±10.7	323.9±31.3	12.39±1.18	75.3	Hairpin
pre-miR-181a-2	AL4	93.0±18.5	271.9±58.6	8.68±0.42	44.3	Duplex
pre-miR-181a-2	AL4	90.0±13.4	267.3±40.4	7.15±0.94	63.7	Hairpin
pre-miR-181a-2		54.7±6.8	168.2±21.3	2.56±0.33	52.2	Hairpin

a – solution: 50 mM NaCl, 50 mM HEPES buffer, 0.5 mM Na_2EDTA, pH 7.5; b - calculated for 10^{-4} M oligomer concentration

Supplementary Table S4. Oligonucleotides used in the study

Oligonucleotide name	Sequence (5′→ 3′) or Catalog and assay number
2′-O-methyl/LNA (in-house)	
AL1	AGAUACCAAACUC
AL2	GCCUUUAGAUACC
AL3	CUCAAACUCACCG
AL4	ACAUUUUUAUACUUUCUC
*h*AL-16-1	AGAAUCUUAACGC
*h*AL16-2	AUUUCACUACGC
Ctrl	CGUAUACUUCGCG
*h*Ctrl	CGUUACGACGAUU
DNA (IBB PAS)	
pre-miR-181a anti-5p arm probe	ACTCACCGACAGCGTTGAATGTT
pre-miR-181a anti-3p arm probe	GGTACAGTCAACGGCCGATGGT
anti-miR-16-5p probe	CGCCAATATTTACGTGCTGCTA
anti-miR-16-1-3p probe	TCAGCAGCACAGTTAATACTGG
anti-miR-16-2-3p probe	TAAAGCAGCACAGTAATATTGGC
RNA (FUTUREsynthesis)	
pre-miR-181a-1	AACAUUCAACGCUGUCGGUGAGUUUGGUAUCUAAAGGCAAAC CAUCGAUCGUUGACUGUACA
pre-miR-181a-2	AACAUUCAACGCUGUCGGUGAGUUUGAGAAAGUAUAAAAAUG UAAACCAUCGGCCGUUGACUGUACC
pre-miR-16-1	UAGCAGCACGUAAAUAUUGGCGUUAAGAUUCUAAAAUUAUCU CCAGUAUUAACUGUGCUGCUGA
pre-miR-16-2	UAGCAGCACGUAAAUAUUGGCGUAGUGAAAUAUAUAUUAAAC ACCAAUAUUACUGUGCUGCUUUA
Morpholino (GeneTools)	
MO-a1-5p	AGATACCAAACTCACCGACAGCGTT
MO-a2-5p	CTTTCTCAAACTCACCGACAGCGTT
MO-a1-3p	GATCGATGGTTTGCCTTTAGATAC
MO-a2-3p	GGCCGATGGTTTATATTTTTATACT
TaqMan MicroRNA Assay (ThermoFisher)	
miR-181a-5p	Cat# 4427975; 000480
miR-181a-1-3p	Cat# 4440886; 004367
miR-181a-2-3p	Cat# 4440886; 005555

miR-182	Cat# 4427975; 000597
snU6	Cat# 4427975; 001973

REFERENCES

1. Thompson JD, Gibson TJ, Higgins DG. Multiple sequence alignment using ClustalW and ClustalX. Curr Protoc Bioinformatics. 2002 Aug;Chapter 2:Unit 2 3.

2. Liu Z, Wang J, Cheng H, Ke X, Sun L, Zhang QC, et al. Cryo-EM Structure of Human Dicer and Its Complexes with a Pre-miRNA Substrate. Cell. 2018 May 17;173(5):1191-203 e12.

3. Ciechanowska K, Pokornowska M, Kurzynska-Kokorniak A. Genetic Insight into the Domain Structure and Functions of Dicer-Type Ribonucleases. Int J Mol Sci. 2021 Jan 9;22(2).
